# Investigation of iterative deconvolution in ^177^Lu-SPECT imaging for lesion dosimetry

**DOI:** 10.1186/s40658-026-00865-5

**Published:** 2026-04-13

**Authors:** Zachary Ells, Grigory Liubchenko, Mikhail Rumiantcev, Maximilian Scheifele, Angelica Noto, Sophie C. Siegmund, Sandra Resch, Andreas Harbach, Rudolf A. Werner, Matthias Brendel, Gabriel Sheikh, Mathias Zacherl, Guido Böning, Lena M. Unterrainer, Sibylle I. Ziegler, Astrid Delker

**Affiliations:** 1 Department of Nuclear Medicine, LMU University Hospital, LMU Munich, Marchioninistr. 15, 81377 Munich, Germany; 2 Department of Nuclear Medicine and Theranostics, Ahmanson Translational Theranostics Division, David Geffen School of Medicine at UCLA, University of California Los Angeles UCLA, Los Angeles, CA USA; 3 Department of Nuclear Medicine, Hannover Medical School (MHH), Hannover, Germany; 4 German Center for Neurodegenerative Diseases (DZNE) Munich, Munich, Germany; 5 Munich Cluster for Systems Neurology (SyNergy), Munich, Germany; 6 German Cancer Consortium (DKTK), Partner Site Munich, Munich, Germany; 7 German Cancer Research Center (DKFZ), Heidelberg, Germany; 8 Bayerisches Zentrum für Krebsforschung (BZKF), Partner Site Munich, Munich, Germany; 9 The Russell H. Morgan Department of Radiology and Radiological Sciences, Division of Nuclear Medicine and Molecular Imaging, Johns Hopkins School of Medicine, Baltimore, MD USA

**Keywords:** Image processing, ^177^Lu, Dosimetry, Lutetium, Lucy-Richardson, Theranostics, Partial volume correction, Absorbed dose, PSMA, DOTA-TATE

## Abstract

**Aim/introduction:**

Quantitative ^177^Lu-SPECT allows for patient specific dosimetry, but due to the limited spatial resolution absorbed doses (AD) can be underestimated. Implementation of the Lucy-Richardson deconvolution (LRD) algorithm for spill-over correction in PET has been investigated. Therefore, the aim of this study was to extend the potential application of LRD to ^177^Lu-SPECT based tumor dosimetry.

**Materials/methods:**

The NEMA IEC Body Phantom (foreground-to-background ratio 8:1, 237:30 kBq/mL) was measured according to the local imaging and reconstruction protocol. The two main parameters of LRD, sigma and number of iterations, were determined in two steps. First, a matched filter resolution analysis was conducted on the ground truth activity distribution as segmented from the NEMA IEC Body Phantom data to define the sigma of a 3D Gaussian point-spread-function, which describes the system’s spatial resolution. Secondly, using this sigma, a suitable number of LRD iterations was determined by comparing sphere recovery coefficients (RC) and signal-to-noise ratios. The selected parameters were then applied to the reconstructed SPECT series (24, 48, and 72 h post-injection) of 20 patients who received either [^177^Lu]Lu-DOTA-TATE (*n* = 10) or [^177^Lu]Lu-PSMA-I&T (y = 10) treatment, in order to evaluate its impact on AD estimates. Lesion AD from the original reconstruction (OR) and OR + LRD were estimated using MIM SurePlan™ MRT. The AD from OR, OR + LRD, and OR + RC (phantom-based recovery correction based on volume) were compared.

**Results:**

A sigma of 6.0 mm and four iterations resulted in an average improvement of 18.9 ± 4.7% and 17.4 ± 7.6% in the sphere recovery coefficients and the signal to noise ratio, respectively. In total, 98 lesions were evaluated ([^177^Lu]Lu-DOTA-TATE: *n* = 42) ([^177^Lu]Lu-PSMA-I&T: y = 56). For OR + LRD and OR + RC an average increase of 22 ± 12% and 57 ± 36% of tumor AD was found.

**Conclusion:**

OR + LRD increased AD compared to OR, independent of administered radiopharmaceutical and lesion location. This study suggests that implementing LRD may be a promising option for image-based spill-over correction in ^177^Lu-SPECT based dosimetry. Further studies are necessary to investigate the effect of different PVC methods, such as LRD or phantom-based correction factors, on overall uncertainty of lesion ADs.

**Graphical Abstract:**

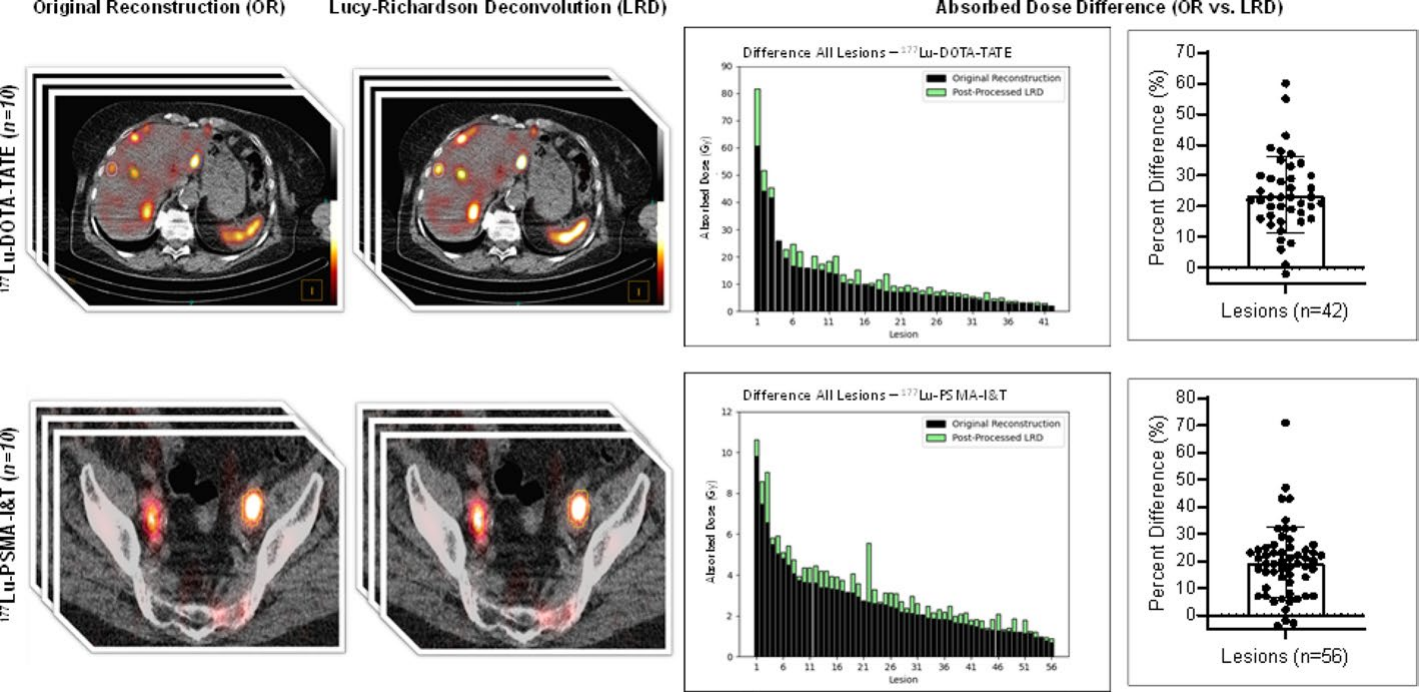

**Supplementary Information:**

The online version contains supplementary material available at 10.1186/s40658-026-00865-5.

## Introduction

A prominent radiopharmaceutical for the treatment of neuroendocrine tumors and prostate cancer is ^177^Lu. In 2018, [^177^Lu]Lu-DOTA-TATE (Lutathera^®^) received approval for treating neuroendocrine tumors [1] following the NETTER-1 Trial. Then, in 2022, [^177^Lu]Lu-PSMA-617 (Pluvicto^®^) was approved as a treatment for metastatic castration-resistant prostate cancer following the VISION Trial [2].

^177^Lu has favorable physical characteristics for clinical applications, which includes a half-life of 6.7 days, a short-range therapeutic β^−^ emission, and gamma ray emissions of 112.9 keV (6.2%) and 208.4 keV (10.4%) useful for gamma camera imaging [3, 4]. Imaging of the ^177^Lu activity distribution at multiple timepoints post-injection using Single-Photon-Emission-Computed-Tomography (SPECT) allows assessment of absorbed doses (AD) to organs-at-risk and lesions, which can be used for individualized treatment planning [5–8]. However, SPECT has a spatial resolution in the order of 5–25 mm (full-width-half-maximum (FWHM)), depending on the employed radiopharmaceutical, collimator, and reconstruction settings [9]. This limited spatial resolution impairs the quantitative accuracy especially in small structures due to activity spill-over (spill-in and spill-out) [10]. This spill-over is also referred to as one cause of partial volume effect (PVE) [11] and can lead to an underestimation of the AD in small structures [9, 12].

Several methods for PVE correction have been investigated for Positron-Emission-Tomography (PET) and SPECT [13, 14]. These approaches include, amongst others, the use of recovery coefficients (RC) derived from phantom measurements, the integration of anatomical information from CT or MRI, or deep learning algorithms [11]. Methods based on artificial intelligence are promising but may be challenging to implement mainly due to a lack of clinical ground truth data. Past studies investigating deconvolution-based algorithms in PET have shown improvement to the quantitative accuracy [15–18]. One example is the Lucy-Richardson deconvolution algorithm (LRD), which was originally developed for improving image quality in optical astronomy [19, 20], but since has been applied in medical imaging. LRD is an iterative statistical deconvolution method, aiming to recover the original, unblurred image. The assumption is that the observed image is a composition of the ground truth image and the system’s point spread function (PSF) describing the image blur due to the limited spatial resolution [12, 19, 20]. Resolution modelling in reconstruction improves quantification in SPECT; however, the primary goal is to restore a homogeneous spatial resolution. Further, the extent to which quantification can be improved in iterative image reconstruction is ultimately limited by the number of iterations that can be performed without introducing excessive noise into the image, given a certain level of regularization.

To expand the full potential of dosimetry for radiopharmaceutical therapies, AD should be as accurate as possible. For ^177^Lu-PRRT or ^177^Lu-PSMA therapy, several dosimetry studies have been reported in which PVE was mitigated by considering lesions above a predefined volume threshold, or use of a recovery coefficient correction factor [21–24]. The aim of this work was to investigate the effect of LRD as a post-processing step on quantitative ^177^Lu-SPECT imaging and image-based tumor dosimetry. Application of LRD will be compared with and without the use of a correction factor derived from phantom-based RC measurements.

## Methods

### Phantom preparation

The NEMA IEC Body Phantom was used to investigate the recovery coefficients (RC) in the six fillable spherical inserts with volumes of 0.5, 1.2, 2.6, 5.6, 11.5, and 26.5 mL, respectively. The phantom was filled with a known ^177^Lu activity concentration ratio (foreground-to-background ratio 8:1 (237:30 kBq/mL). Acquisition and reconstruction parameters were identical to clinical routine as described in forthcoming sections.

### Image acquisition

SPECT data for 208 keV (width: 15%) was acquired on the Siemens Symbia T2 equipped with a medium-energy collimator (Siemens Healthineers, Erlangen, Germany). 64 projections per detector head were measured with 5 s measurement time per projection and a matrix of 128$\:\times\:$128 pixels [25]. For patient CT imaging, CareDose was enabled using a reference tube current of 15 mAs and 110 kV tube voltage.

### Quantitative SPECT reconstruction

SPECT images were reconstructed using Hermes Hybrid Recon-Oncology 4.0 (Hermes Medical Solutions, Sweden) and the maximum-a-posteriori ordered-subset expectation–maximization algorithm (OSEM MAP-Smooth, β = 0.001, 16 iterations/8 subsets) [26]. Quantitative reconstruction included CT-based attenuation correction, Monte Carlo-based scatter correction, and resolution modeling [27].

### Recovery coefficient and signal-to-noise ratio

Spherical volume-of-interest (VOI)s with known volume were placed on the phantom spheres using the high-resolution CT (voxel size: 0.98$\:\times\:$0.98$\:\times\:$3 mm³). The average activity concentration was measured using HERMES Affinity 3.0.5 (Hermes Medical Solutions, Sweden). RCs were then determined by dividing the measured activity concentration in the pre-defined VOIs by the known activity concentration. Further, four VOIs were placed throughout the background compartment of the phantom to determine the mean background activity concentration and the corresponding standard deviation, as required to calculate the signal-to-noise ratio (SNR) for each sphere.

Volume-dependent RCs were modelled by a the following fit function: $\:{f}_{RC}\:\left(V\right)=1-\:{\left(1+{\left(\frac{V}{b}\right)}^{y}\right)}^{-1}$ where V represents the volume, while b and y are fitting parameters was used [28–30].

### Lucy-Richardson deconvolution

As described, LRD is a statistical iterative deconvolution method assuming that the current image estimate is a convolution of the original image with the system’s PSF. The PSF was assumed to be a symmetric and spatially invariant 3D Gaussian function, where sigma was defined via a matched filter resolution analysis (MFA) as described prior [31, 32]. Briefly, a 3D ground truth activity distribution was derived from the segmented CT of the NEMA IEC body phantom. The ground truth image was then filtered with a 3D Gaussian, where sigma was successively increased in steps of 0.48 mm in the range of 0–48 mm. The sigma providing the minimum root mean squared error (RMSE) between the post-filtered ground truth image and the original reconstruction of the NEMA IEC body phantom was considered as the systems spatial resolution. The RMSE was calculated based on the NEMA IEC body phantom spheres with volumes of 2.6, 5.6, 11.5, and 26.5 mL. The smallest two spheres, 0.5 and 1.2 mL, were excluded as they were non-visible in the SPECT data.

Using the given acquisition, the assumption is our data follows Poisson statistics; accordingly LRD uses a maximum likelihood expectation maximization approach, therefore applying the appropriate number of iterations is key for the algorithm. A high number of iterations will lead to higher RCs, but also increases noise and decreases the signal-to-noise ratio. Therefore, number of iterations was selected by investigating the relationship between RC and SNR in the NEMA IEC body phantom. Therefore, LRD was applied on the reconstructed 3D-SPECT data using the pre-determined PSF as described prior, with varying sigma and number of iterations. Implementation of the algorithm is available in Python (version 3.11.0) package scikit-image (Version 0.23.2) [33].

### Patient cohort

Two patient cohorts with 10 patients each were selected to test the effect of LRD on tumor dosimetry. The first 10 patients presented with neuroendocrine tumors and received [^177^Lu]Lu-DOTA-TATE (average ± standard deviation: 7308 ± 132 MBq), while the second group presented with metastatic castration-resistant prostate cancer and received [^177^Lu]Lu-PSMA-I&T (7499 ± 162 MBq). The two diseases were selected to obtain a broad variation in the tumor uptake in relation to the background activity level, different tumor volumes, and diverse locations. Only the first therapy cycle was considered in this analysis.

According to the regulations of the German Pharmaceuticals Act § 13(2b), all patients gave written consent to undergo treatment. This analysis was performed in compliance with the principles of the Declaration of Helsinki and was approved by the institutional ethics board of the LMU Munich (24–0982).

### Lesion selection and segmentation

Lesions must have been visible on SPECT at all three imaging timepoints to be included in analysis. Two different segmentation methods were employed on the SPECT acquired at 24 h post-injection, based on the therapy received. The PET Edge+ tool available in MIM (GE Healthcare's MIM Software Inc., Cleveland, OH; version 7.3.4) was used in the segmentation of the post-therapy SPECT after [^177^Lu]Lu-DOTA-TATE. For, patients who received [^177^Lu]Lu-PSMA-I&T therapy, the single click segmentation tool available in HERMES Affinity (Hermes Medical Solutions, Stockholm, Sweden; version 3.0.5) was used with an SUVmax threshold of 3.0 as suggested by John et al. [34].

The tumor-to-background ratio was independently determined for each lesion; background activity was determined by placing four identical VOIs of the tumor just outside the tumor boundaries and taking the average.

### Absorbed dose calculation

Tumor dosimetry was based on multiple timepoint SPECT imaging at 24, 48, and 72 h post-injection. Dosimetry calculations were performed using MIM SurePlan™ MRT (version 7.3.4) using the original SPECT reconstruction (OR) and the OR with subsequent LRD (OR + LRD). Lesion segmentations were defined on the OR, however differences in the segmented lesion volumes based on OR + LRD were evaluated. Registration of individual volumes of interest between timepoints was visually verified. Voxel-wise time-activity data was fit using a monoexponiental function. Conversion of voxel-wise time-integrated activities (TIA) was then performed via convolution with a voxel S-value kernel [35]. Mean tumor ADs derived from the OR, OR + LRD, and OR corrected by a RC-based, volume-derived (OR + RC) correction factor as derived from the aforementioned NEMA IEC body phantom were compared.

## Results

### Phantom measurement and evaluation

**Fig. 1:**
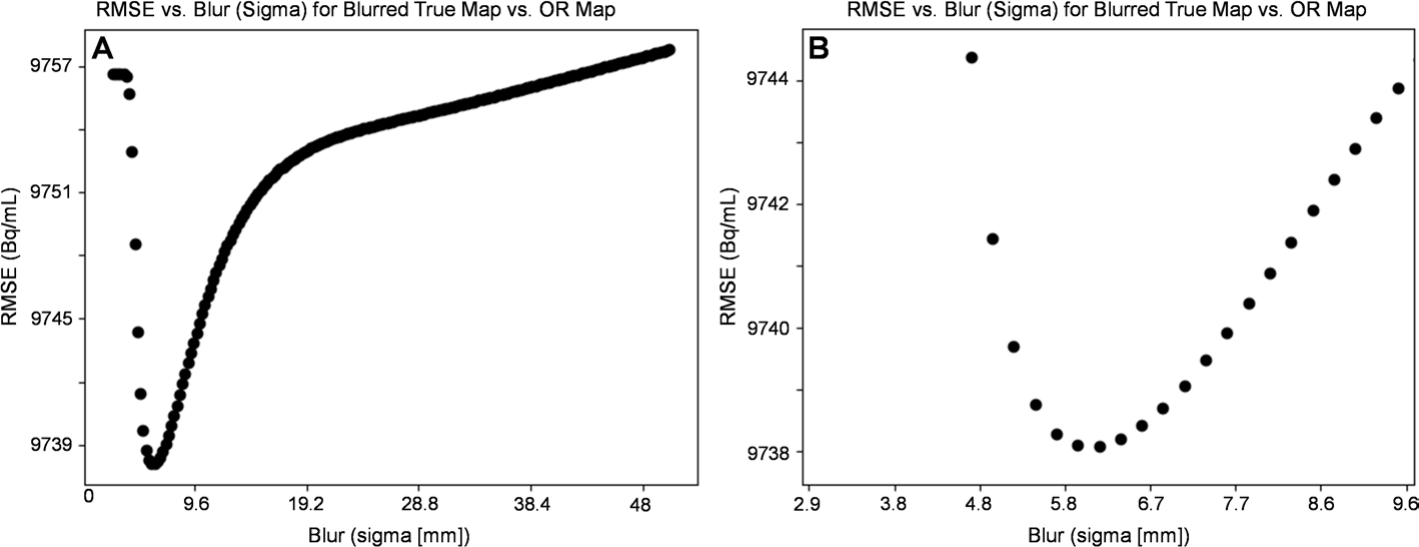
Initial determination of sigma and number of iterations to optimize the LRD algorithm. (**A**) RMSE over all tested sigmas, (**B**) magnified, showing sigma of 6.0 mm is optimal

**Fig. 2:**
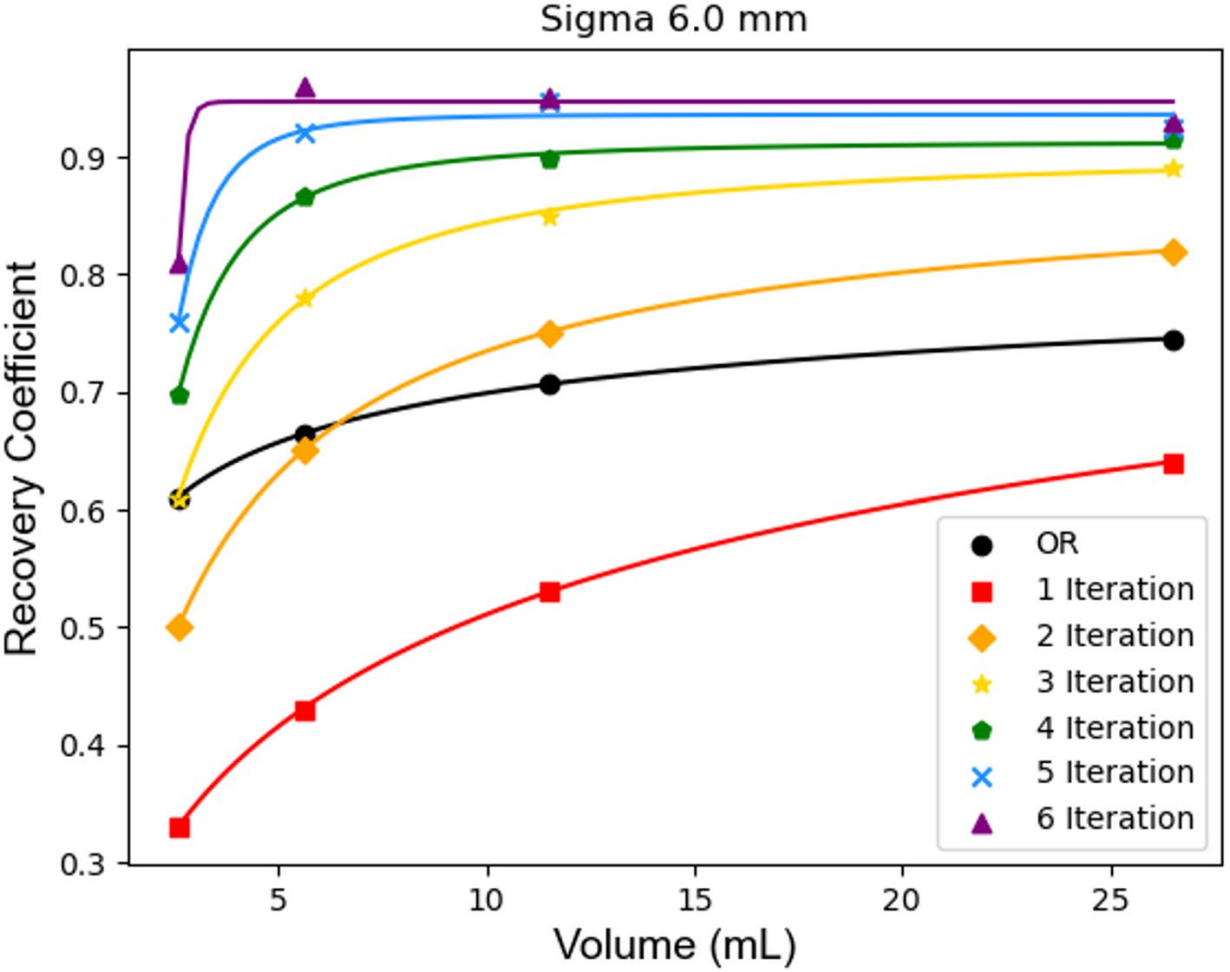
RCs in each sphere with varying number of iterations applied to LRD using a sigma of 6.0 mm

According to the MFA, the FWHM characterizing the reconstructed spatial resolution was found to be 14.1 mm (sigma: 6.0 mm) (Fig. 1A and B). Corresponding RCs and SNRs can be found in Table 1 on a per LRD iteration basis. Results for the two smallest spheres are not presented as the activity was not visible in the SPECT images, and not used in determination of LRD parameters. Increasing number of iterations of LRD resulted in the RCs increasing and SNRs decreasing (Fig. 2; Table 1). The percent gain in RC and SNR both decrease as the number of LRD iterations increases. Due to this observation in RC and SNR we opted for 4 iterations. Supplemental Fig. 1 shows LRD applied (sigma 6.0 mm) up to 12 iterations. Total activity in the entire field-of-view was preserved. Evaluation of algorithm under various foreground-to-background ratios, reconstruction parameters, and image acquisition settings is shown in the supplemental Tables 1–3.

**Table 1:** Phantom RCs and SNRs based on the OR and sigma (S) + number of iterations (i)

	Recovery coefficient				Signal to noise ratio			
	2.6 mL	5.6 mL	11.5 mL	26.5 mL	2.6 mL	5.6 mL	11.5 mL	26.5 mL
OR	**0.61**	**0.66**	**0.71**	**0.75**	**7.05**	**8.60**	**10.90**	**10.95**
S6.0 mm + 1i	0.33	0.43	0.53	0.64	12.17	16.07	20.09	24.29
S6.0 mm + 2i	0.50	0.65	0.75	0.82	8.97	12.55	14.78	16.57
S6.0 mm + 3i	0.61	0.78	0.85	0.89	9.02	12.07	13.23	13.96
**S6.0 mm + 4i**	**0.70**	**0.87**	**0.90**	**0.91**	**9.01**	**11.61**	**12.15**	**12.39**
S6.0 mm + 5i	0.76	0.92	0.93	0.93	9.02	11.25	11.40	11.39
S6.0 mm + 6i	0.81	0.96	0.95	0.93	9.01	11.05	11.38	11.22

Bold text represents the original reconstruction while the bold underline text represents the selected sigma and number of iterations used in the study based on MFA, RC, and SNR.

### Patient lesions

Detailed information regarding the patients and the lesions selected can be found in Table 2. AD per therapy and per lesion location can be found in Table 3. Patients presented with 1 to 8 lesions. 78/98 (80%) lesions were < 10 mL. Although segmentations were based on the OR for AD calculations, the difference in volume was always within 3% when segmented on the OR + LRD images.

**Table 2:** Patient cohort characteristics

Characteristic	
[^177^Lu]Lu-DOTA-TATE	
Patients	10
Gender (m/f)	4/6
Total Lesions	42
Lesions per patient	
Median [range]	5 [1–7]
Liver Lesions	34
Average Volume (mL)	10.5 ± 29.8
Median [range] Volume (mL)	3.6 [1.6–175.5]
Lymph Node Lesions	8
Average Volume (mL)	46.3 ± 77.4
Median [range] Volume (mL)	20.67 [0.5–232.2]
[^177^Lu]Lu-PSMA-I&T	
Patients	10
Gender (m/f)	10/0
Total Lesions	56
Lesions per patient	
Median [range]	6 [1–8]
Bone Lesions	35
Average Volume (mL)	6.0 ± 11.8
Median [range] Volume (mL)	2.2 [0.7–60.7]
Lymph Node Lesions	20
Average Volume (mL)	11.2 ± 14.9
Median [range] Volume (mL)	4.1 [1.5–64.2]
Soft Tissue Lesion	1
Volume (mL)	4.6

**Table 3:** Dosimetry differences between OR, LRD, and OR + RC

Dosimetry	Absorbed doses per unit activity (Gy/GBq)		
	OR	OR + LRD	OR + RC
*[* ^*177*^ *Lu]Lu-DOTA-TATE*			
Liver Lesions (n_1_ = 34)			
Average ± SD	11.1 ± 13.1	14.1 ± 16.4	16.9 ± 17.9
[Range]	[1.7–60.7]	[2.0–81.7]	[2.4–84.2]
Lymph Node Lesions (n_2_ = 8)			
Average ± SD	11.6 ± 7.0	13.4 ± 6.0	24.9 ± 21.5
[Range]	[4.0–25.8]	[7.0–25.2]	[9.3–73.7]
*[* ^*177*^ *Lu]Lu-PSMA-I&T*			
Bone Lesions (y_1_ = 35)			
Average ± SD	2.4 ± 1.7	2.8 ± 1.8	6.0 ± 4.2
[Range]	[0.7–9.8]	[0.9–10.6]	[1.44–22.7]
Lymph Node Lesions (y_2_ = 20)			
Average ± SD	3.2 ± 1.7	4.1 ± 2.1	4.8 ± 2.3
[Range]	[1.2–7.5]	[1.8–9.0]	[2.2–10.4]
Soft Tissue Lesion (y_3_ = 1)			
	1.6	2.1	2.4

**Fig. 3:**
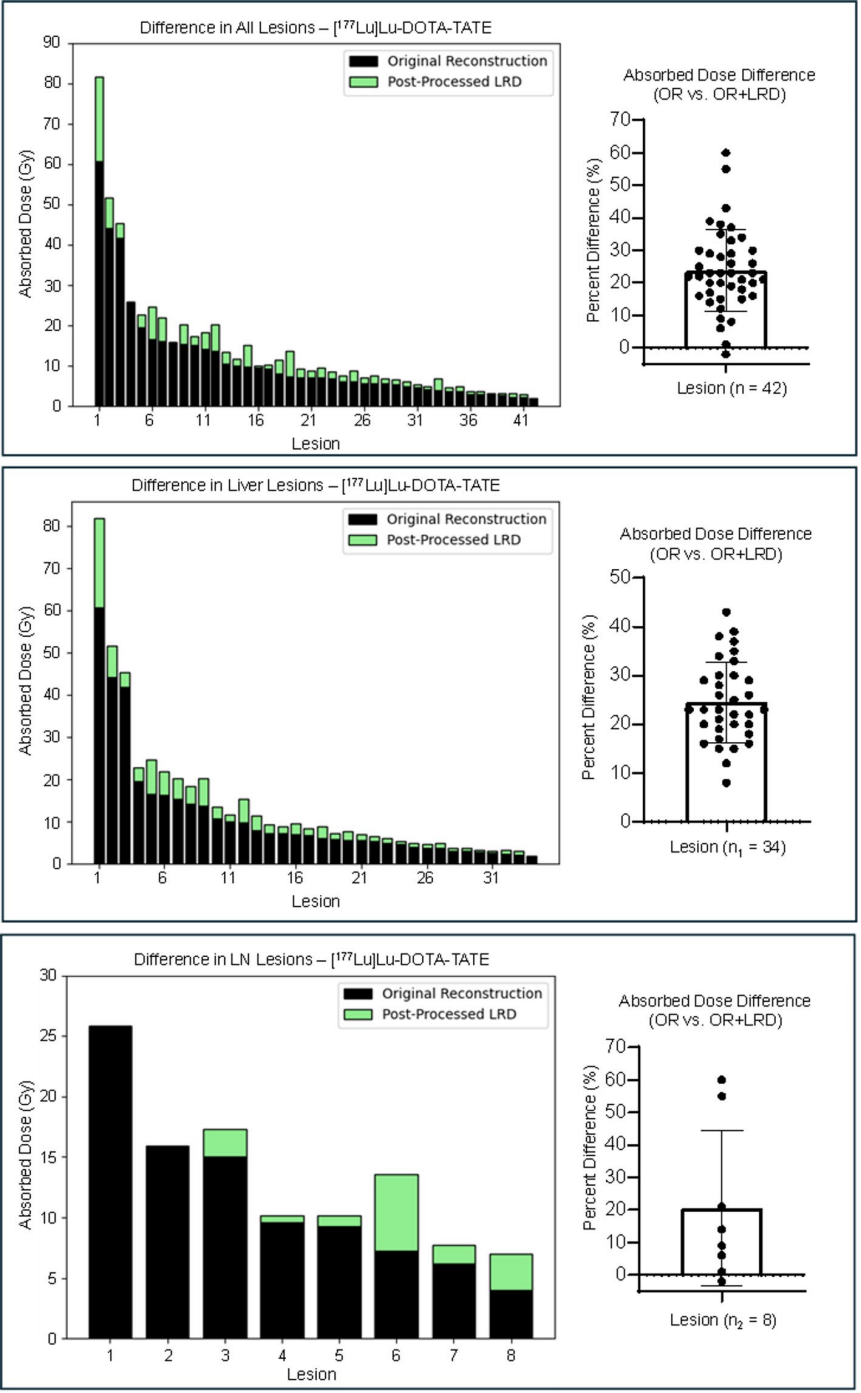
Comparison of lesion AD from [^177^Lu]Lu-DOTA-TATE treatment without (black) and with (green) LRD. AD always increased when using OR + LRD in comparison to OR except in one liver lesion case (< 1% difference)

**Fig. 4:**
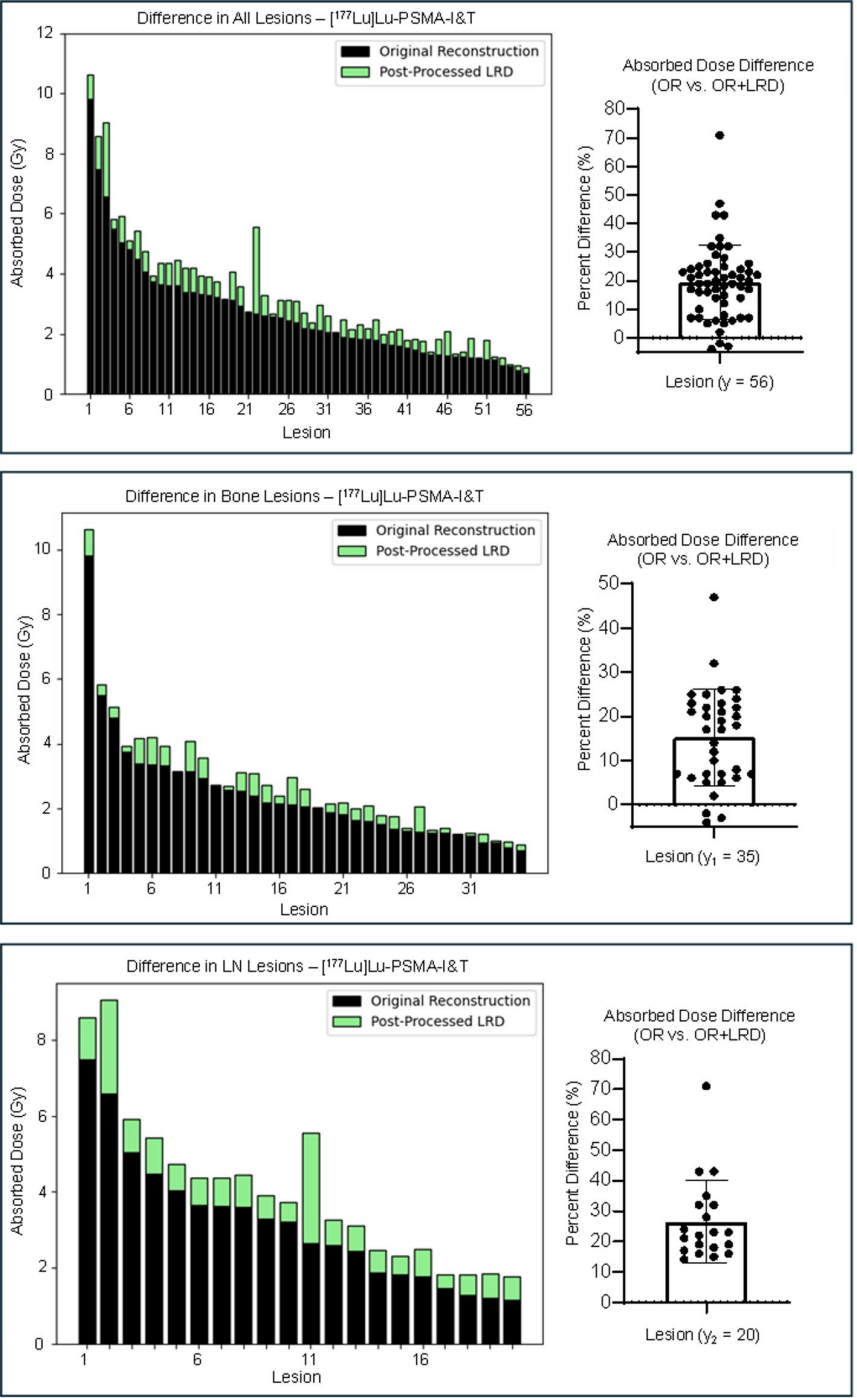
Comparison of lesion AD from [^177^Lu]Lu-PSMA-I&T treatment without (black) and with (green) LRD. AD always increased when using OR + LRD in comparison to OR except in three bone lesion cases (< 3% difference) in a single patient

In total there was an average percent difference of 22 ± 12% between the calculated AD from OR and OR + LRD (Table 4; Figs. 3 and 4). In 14/98 (14%) lesions, the AD difference from OR and OR + LRD only varied by 5–10%. In 80/98 (82%) lesions, the AD calculated using OR + LRD were about 20% greater, regardless of the patient cohort, or lesion location. In 4/98 (4%) lesions, using the OR resulted in higher AD. These cases were specific to two patients, one was from a 47 mL liver lesion in a patient receiving [^177^Lu]Lu-DOTA-TATE, while the other three cases were bone lesions with volumes of 2.7, 2.2, and 1.9 mL from a patient receiving [^177^Lu]Lu-PSMA-I&T. In all four cases, though, the absolute percent difference between the OR and OR + LRD was within 3%. Table 4 summarizes the results based on OR + LRD and OR + RC compared to OR. The supplemental Table 4 shows the activity quantification (Bq/mL) difference between LRD and OR for each timepoint, and cohort evaluated.

**Table 4:** Absorbed doses from LRD and OR + RC compared to OR

Average absolute percent difference (%±SD%) from OR absorbed doses		
	OR + LRD	OR + RC
Overall (*n* = 98)	22 ± 12	57 ± 36
[^177^Lu]Lu-DOTA-TATE		
Total Lesions (*n* = 42)	24 ± 12	47 ± 25
Liver Lesions (n_1_ = 34)	25 ± 8	46 ± 14
Lymph Node Lesions (n_2_ = 8)	21 ± 7	53 ± 51
[^177^Lu]Lu-PSMA-I&T		
Total Lesions (y = 56)	20 ± 12	65 ± 41
Bone Lesions (y_1_ = 35)	23 ± 14	77 ± 46
Lymph Node Lesions (y_2_ = 20)	15 ± 10	44 ± 17
Soft Tissue Lesion (y_3_ = 1)	29	38

## Discussion

The PVE is a known limitation with SPECT imaging as it causes a size-dependent loss in quantitative accuracy. Thus, this exploratory study investigates the effects of implementing LRD in ^177^Lu lesion dosimetry. Using LRD resulted in an increase in lesion AD in 94/98 (96%) cases, with an average increase of 22 ± 12%. While many publications report AD to tumors, few account for PVE correction with regards to ^177^Lu-PSMA [36, 37], which - if omitted - can lead to underestimation of calculated AD. A few studies report promising absorbed dose relationships for ^177^Lu-PRRT where PVE was addressed by applying a RC-derived correction factor or applying a lesion volume threshold for AD evaluation [21–23]. Either scenario strengthens the value of adding a PVE correction to AD estimates.

Phantom data can be used to derive simple correction factors [37, 38]. The application of RCs (OR + RC) led to an increase of 57 ± 36% for lesion AD, however, this approach has its limitations in clinical practice as lesions are subject to a variable geometry and tumor-to-background ratios, both leading to a varying lesion recovery [39, 40]. Further, the positioning of objects relative to each other influences the actual object recovery [41]. As a methodological improvement, Marquis et al. considered the influence of the actual object shape within RC-based PVC [42]. Still, when using OR + RC, foreground-to-background ratios similar to the ones used in the phantom measurements are assumed (in our case we used 8:1: (237:30 kBq/mL)). However, in reality this tumor-to-background ratio is unique to each lesion.

**Fig. 5:**
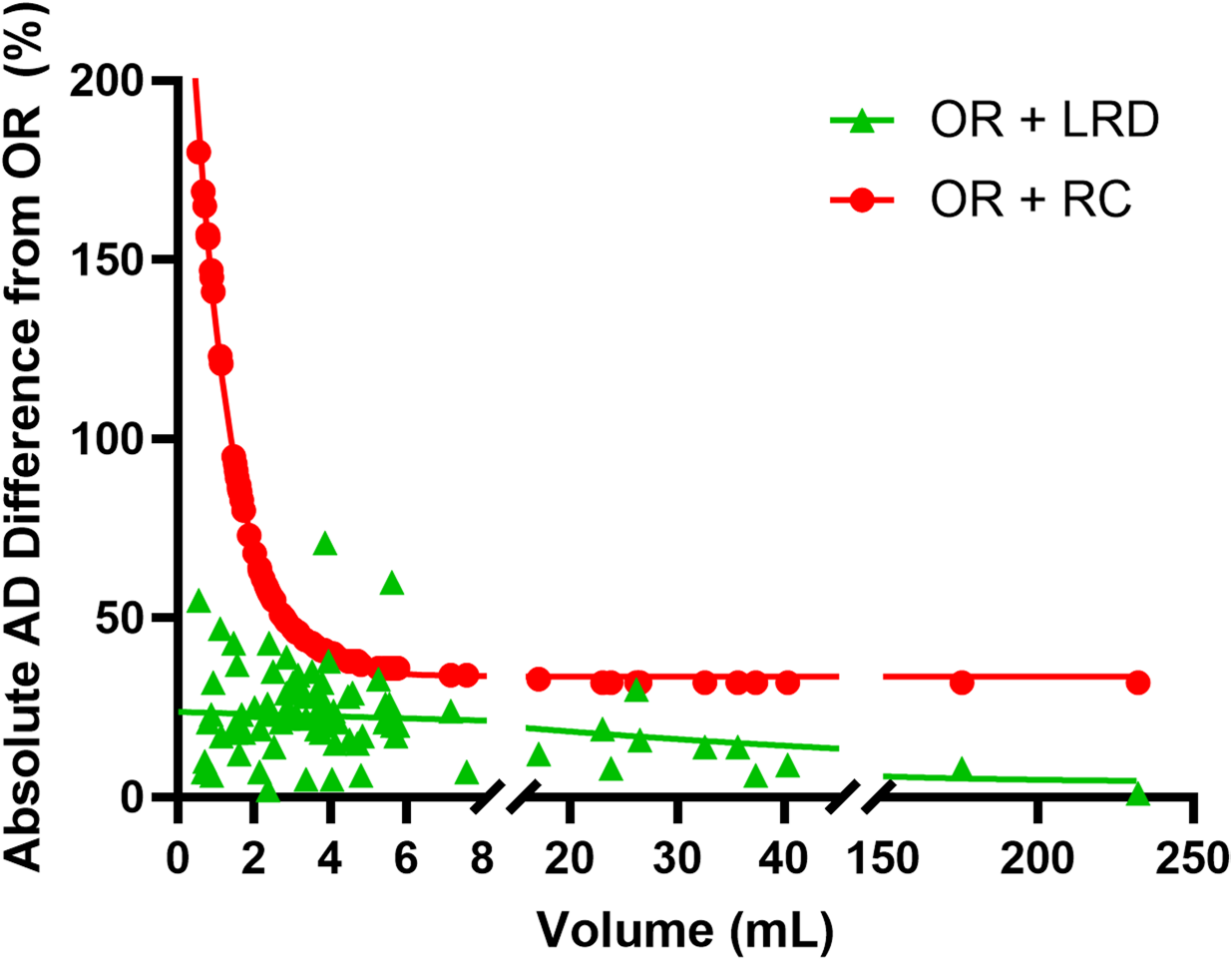
Percent difference in the AD from OR + LRD and OR + RC in all lesions. Convergence of the RC curve is extremely slow for OR + RC as shown through the stabilization around 40% for large volumes

**Fig. 6:**
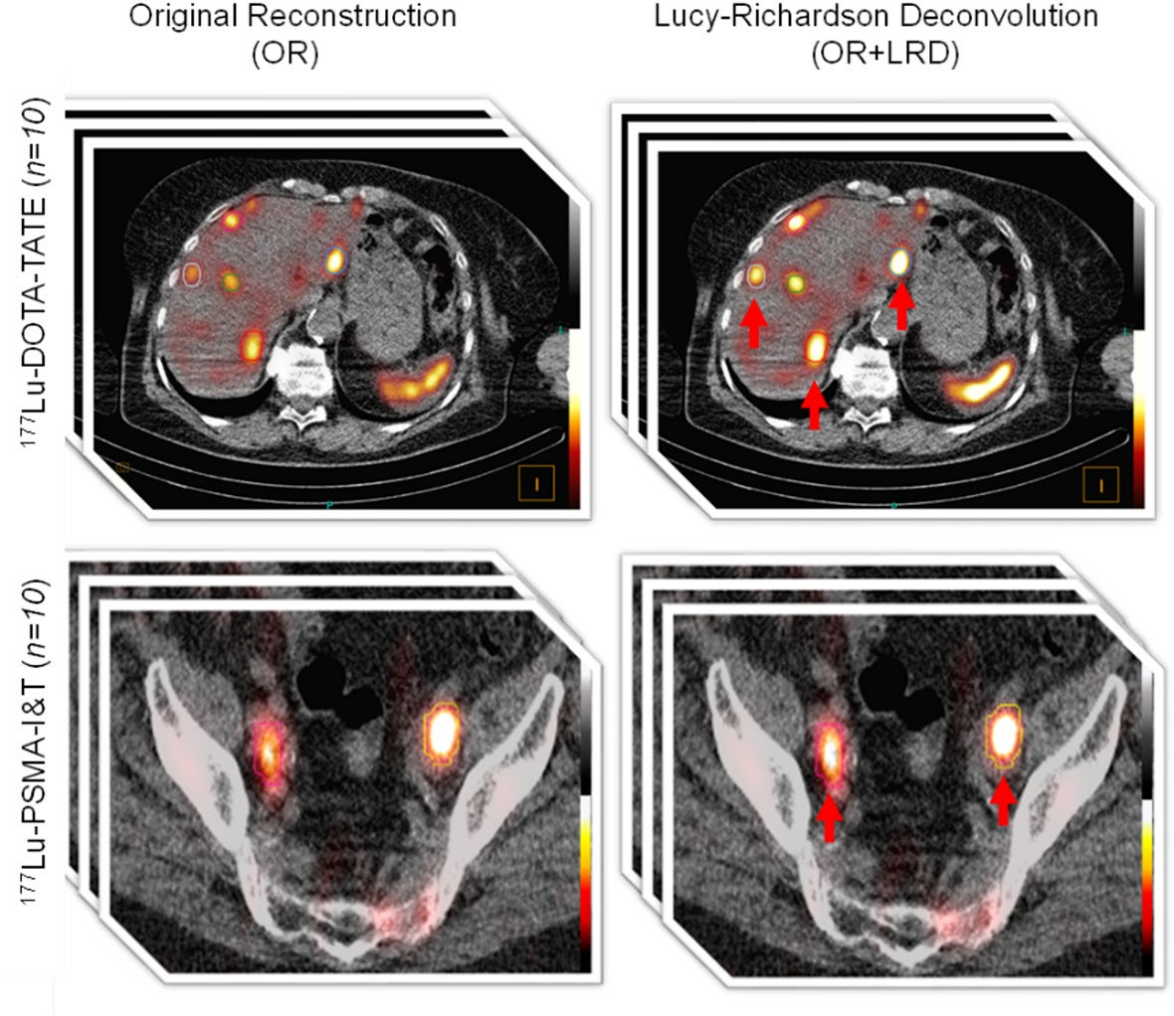
Exemplary SPECT/CT data for both patient cohorts acquired at three time points post injection with and without LRD applied. Red arrows indicate selected lesions

The PVE correction difference as a function of lesion size depending on the use of OR + LRD or OR + RC can be found in Fig. 5. When using OR + RC, small volume lesions receive higher corrections, therefore resulting in higher AD. The lower absorbed doses from LRD compared to OR + RC could potentially be due to the fact that LRD does not reach full recovery when applied to the phantom data; therefore it still results in an underestimation of tumor absorbed doses. In this study, it is unfortunately not possible to compare OR + LRD and OR + RC in the clinical data set as the ground truth lesion AD is not known. The application of LRD in activity quantification in patient-like digital phantoms has been reported in a previous publication, showing that LRD increased median recovery, although they remained below 100% [43]. In contrast to OR + RC, LRD is an image-based approach simple in its application as a post-processing, and acts on the voxel level without the assumption of a specific geometry but depends on a sophisticated parameter settings. Example patient images comparing OR and OR + LRD can be seen in Fig. 6.

The PSF was assumed to follow a spatially invariant 3D Gaussian function, which was defined based on a blurred ground truth image of the NEMA IEC body phantom. Even though PSF-modelling in quantitative SPECT reconstruction seeks to enhance and homogenize spatial resolution, convergence slows and is object-dependent, thereby altering the local PSF to be position-dependent. Hence, achieving perfect resolution compensation remains a challenge.

Liu et al. used the reblurred Van-Cittert algorithm [44] and Iterative Yang in comparison to RCs to address PVE in simulated and clinical data in the context of SPECT imaging for [^177^Lu]Lu-PSMA therapy [14, 45]. The reblurred Van-Cittert employs iterative deconvolution of the system’s PSF like LRD, while Iterative Yang considers the system’s PSF with combination of anatomical knowledge (i.e. appropriate segmentation). Liu et al. observed an increase of 63.5 ± 25.2% and 82.3 ± 33.5% in 40 clinically evaluated lesions using the reblurred Van-Cittert and RCs respectively [45]. This average increase is higher compared to the results in this study, but could be partially attributed to a lower initial RC in the study by Liu et al. The non-corrected RC for the largest investigated sphere of 40 mm was reported to be 70% (foreground-to-background ratio 10:1) [45], while the RC for the largest sphere of 37 mm (26.5 mL) in this study was 75% (foreground-to-background ratio of 8:1). Further, while the PSF was determined based on a MFA in this study, Liu et al. used a simulated and reconstructed line source. However, the results published by Liu et al., and in this investigation demonstrate that deconvolution-based PVE correction can assist in more accurate quantification and enhanced visualization.

In cases where anatomical knowledge is known (e.g. kidneys or salivary glands) implementing the Iterative Yang [11] method could also be an alternative option to solely PSF-based iterative deconvolution [45]. However, for lesion dosimetry this approach is highly limited, as an accurate anatomical segmentation is usually not available. Additionally, the required segmentation makes Iterative Yang more time consuming and potentially prone to errors compared to LRD or reblurred Van-Cittert. Leube et al. implemented a PVE correction based on deep learning for ^177^Lu-SPECT imaging based on a dataset of 10,000 simulated and random activity distributions, providing improved SPECT image quality and quantification which also outperformed Iterative Yang [41]. Hammersen et al. systematically investigated an oversize-volume of interest approach for the quantification of lesion uptake in the context of reliable pretherapeutic PET-based dosimetry [46].

There are several limitations to our study that needs to be stated, the first is that while the patient cohort included was designed purposefully to be heterogeneous based on tumor presentation (variable tumor to background ratios, location, and volumes), it was nevertheless a small total population (*n* = 20). Secondly, the RC determined was based on a single sphere-to-background (8:1) used as a representative function across all tumors in the study. The average tumor-to-background ratio of the included lesions was 9.2. Also, LRD parameters were determined based on a single phantom measurement with sphere-to-background of 8:1. Sigma and number of iterations for LRD are crucial to balance image quantification and noise amplification. Supplemental Figs. 2–5 show line profiles of the phantom with OR and OR + LRD, example phantom images, and example patient images. Supplemental Tables 1–3 vary image acquisition, reconstruction, and foreground-to-background ratios to evaluate LRD parameters, but deeper investigations on methodological implementation should be completed. Sites should define LRD parameters independently based on own clinical acquisition protocol, reconstruction settings, and clinically relevant sphere-to-background ratios.

Future tasks should focus on comparing available approaches for PVE correction in SPECT in terms of accuracy, clinical feasibility, and robustness for individualized treatment planning. Furthermore, evaluating the behavior for a range of reconstruction and acquisition settings would be valuable in the complete analysis of LRD.

## Conclusion

Implementation of LRD into the tumor AD calculation for ^177^Lu-based therapies can assist in the PVE correction. LRD is a simple-in-use option that resulted in an increase in lesion AD in 94/98 (96%) cases, with an average increase of 22 ± 12%, meanwhile recovery coefficient based corrections resulted in an increase of 57 ± 36%.

## Supplementary Information

Below is the link to the electronic supplementary material.

Supplementary Material 1.

## Data Availability

Please contact the corresponding author.

## Abbreviations

OR: Original reconstruction
LRD: Lucy-Richardson deconvolution
RC: Recovery coefficients
OR + RC: Original Reconstruction with Recovery Coefficient Correction Applied
RMSE: Root mean squared error
AD: Absorbed dose
MFA: Matched filter resolution analysis
SNR: Signal to noise ratio
