# Supplementary material for: Investigation of iterative deconvolution in 177Lu-SPECT imaging for lesion dosimetry
Source: EJNMMI Phys. 2026 Apr 13;13:34. doi: 10.1186/s40658-026-00865-5 (PMC13076692; doi:10.1186/s40658-026-00865-5)

**Recovery Coefficient per Sphere vs. LRD Iteration**


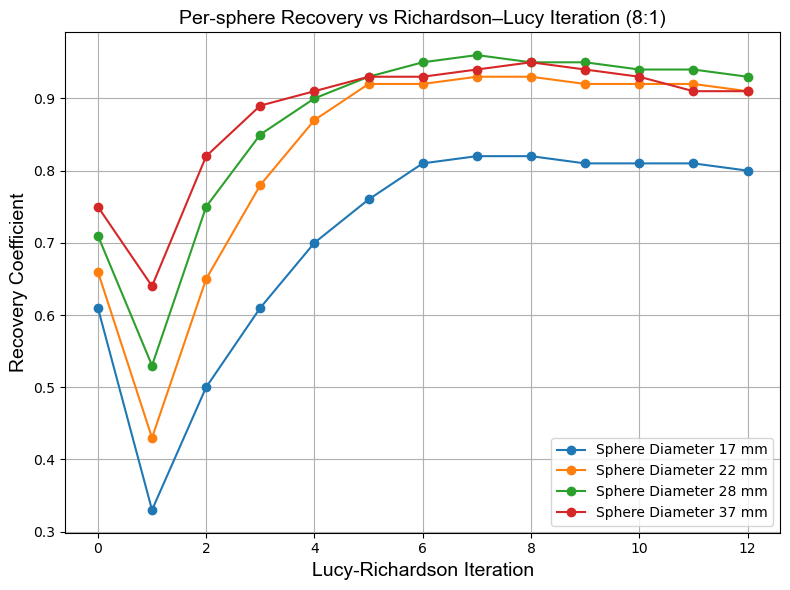


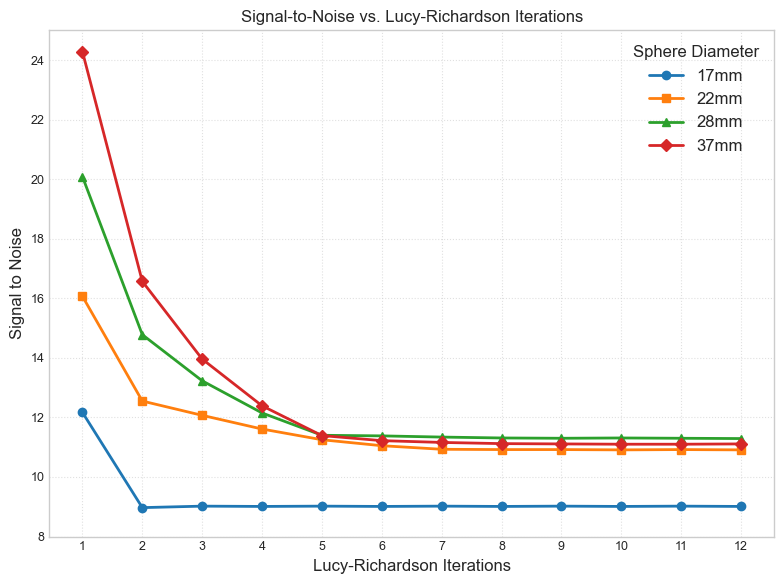


Figure 1: The 4 largest NEMA IEC body phantom spheres as a function of number of iterations of LRD implemented and RC (TOP) and SNR (BOTTOM)

**Influences of Acquisition and Reconstruction on LR parameters**

SPECT acquisition of images were completed at each institution using unique parameters.

Table 1: Acquisition parameter differences between universities.

| Acquisition Parameters | Ludwig Maximilians U. | U. California Los Angeles |
| --- | --- | --- |
| Method | 5s Continuous | 10s Continuous |
| Matrix Size | 128 x 128 | 128 x 128 |
| Photopeak [keV] width (%) | 208 (15) | 208 (15) |
| Scatter Window Correction energy [keV] width (%) | 170 (15), 240 (10) | 170 (15), 240 (10) |

The raw tomographic data with the corresponding CT was then reconstructed using the unique protocol seen below:

Table 2: Reconstruction parameter differences between universities

| Reconstruction Parameters | |
| --- | --- |
| Ludwig Maximilians U. | U. California Los Angeles |
| MAP Smoothing - OSEM | OSEM |
| 16 iterations 8 subsets | 48 iterations 1 subset |
| No Post-Filtration | No Post-Filtration |
| β = 0.001 | β = 0 |

**Optimal Sigma Parameter for LR Over the Whole Image**

Robustness evaluation of LRD parameter optimization. All patient data presented originated from LMU. Phantom acquisition from UCLA sole purpose was for algorithm robustness evaluation.

Table 3: Evaluation of LRD parameters over varying acquisition and reconstructions with corresponding FWHM. Reconstructions completed using HERMES Hybrid Recon 4.0,

| Acquisition | Reconstruction | Sphere:Bkg (kBq/mL) | Ratio | Sigma | Iterations | FWHM |
| --- | --- | --- | --- | --- | --- | --- |
| LMU | 16i8s β0.001 | 1050:70 | 16:1 | 7.20 | 2 | 17.0 |
| UCLA | 48i1s | 2056:130 | 16:1 | 6.40 | 5 | 14.7 |
| UCLA | 16i8s β0.001 | 2056:130 | 16:1 | 6.30 | 4 | 14.5 |
| LMU* | 16i8s β0.001 | 293:30 | 8:1 | 6.00 | 4 | 14.1 |
| LMU | 48i1s | 293:30 | 8:1 | 6.20 | 4 | 14.3 |
| LMU | 8i8s β0.001 | 620:80 | 8:1 | 7.20 | 2 | 17.0 |
| LMU | 16i8s β0.001 | 620:80 | 8:1 | 6.60 | 2 | 15.5 |
| LMU | 4i8s β0.001 | 620:80 | 8:1 | 8.40 | 2 | 19.8 |
| LMU | 16i8s β0.01 | 620:80 | 8:1 | 6.60 | 4 | 15.5 |
| LMU | 16i8s β0.001 | 270:70 | 4:1 | 7.80 | 3 | 18.4 |

*Implemented in the clinical evaluation

**Activity Concentration (Bq/mL) Difference**

Table 4: Activity concentration in lesions from both therapies between the OR and LRD at each timepoint

|  | 24 hr SPECT/CT | | 48 hr SPECT/CT | | 72 hr SPECT/CT | |
| --- | --- | --- | --- | --- | --- | --- |
|  | Average ± SD | Median | Average ± SD | Median | Average ± SD | Median |
| **Total Lesions** | **153502 ± 184252** | **94852** | **99151 ± 135663** | **67812** | **81359 ± 126303** | **47182** |
| DOTA - ALL | 237910 ± 208546 | 169687 | 143138 ± 181574 | 112428 | 127749 ± 168437 | 88994 |
| DOTA - LIVER | 265969 ± 219266 | 185482 | 179635 ± 153672 | 127968 | 166515 ± 143215 | 117114 |
| DOTA - LN | 118660 ± 89954 | 78535 | -11976 ± 218696 | 59213 | -37008 ± 176629 | 23779 |
| PSMA - ALL | 90197 ± 133909 | 69916 | 66161 ± 72787 | 45097 | 46556 ± 63811 | 31350 |
| PSMA - BONE | 70445 ± 58936 | 65402 | 65305 ± 62974 | 47842 | 46624 ± 55555 | 31425 |
| PSMA - LN | 125907 ± 208870 | 76886 | 69723 ± 90152 | 44352 | 45229 ± 78929 | 30813 |

When evaluating the data with this respect there are several points to acknowledge but are consistent with the absorbed dose analysis.

Regarding DOTA: As shown in the manuscript figure 3b (Lymph node lesions) there were 2 cases in which the lesion absorbed dose difference between OR and LRD was near, or below zero. This evaluation uncovered that in timepoint 2 and 3 in these two lesions the activity concentration was greater from OR compared to LRD.

Regarding PSMA: As shown in the manuscript figure 4b (bone lesions) there were 4 cases in which the lesion absorbed dose difference between OR and LRD was near, or below zero. The 3 cases that the OR activity concentration was greater than LRD it was at timepoint 1 and 2; timepoint 1 and 3; and timepoint 1 respectively. Additionally there were 4 instances at a single timepoint in which the activity concentration between OR and LRD was greater in the former, or nearly identical. In all 7 cases these lesions had the lowest difference between OR and LRD as shown in figure 4b.

**Line Profiles within Phantom**


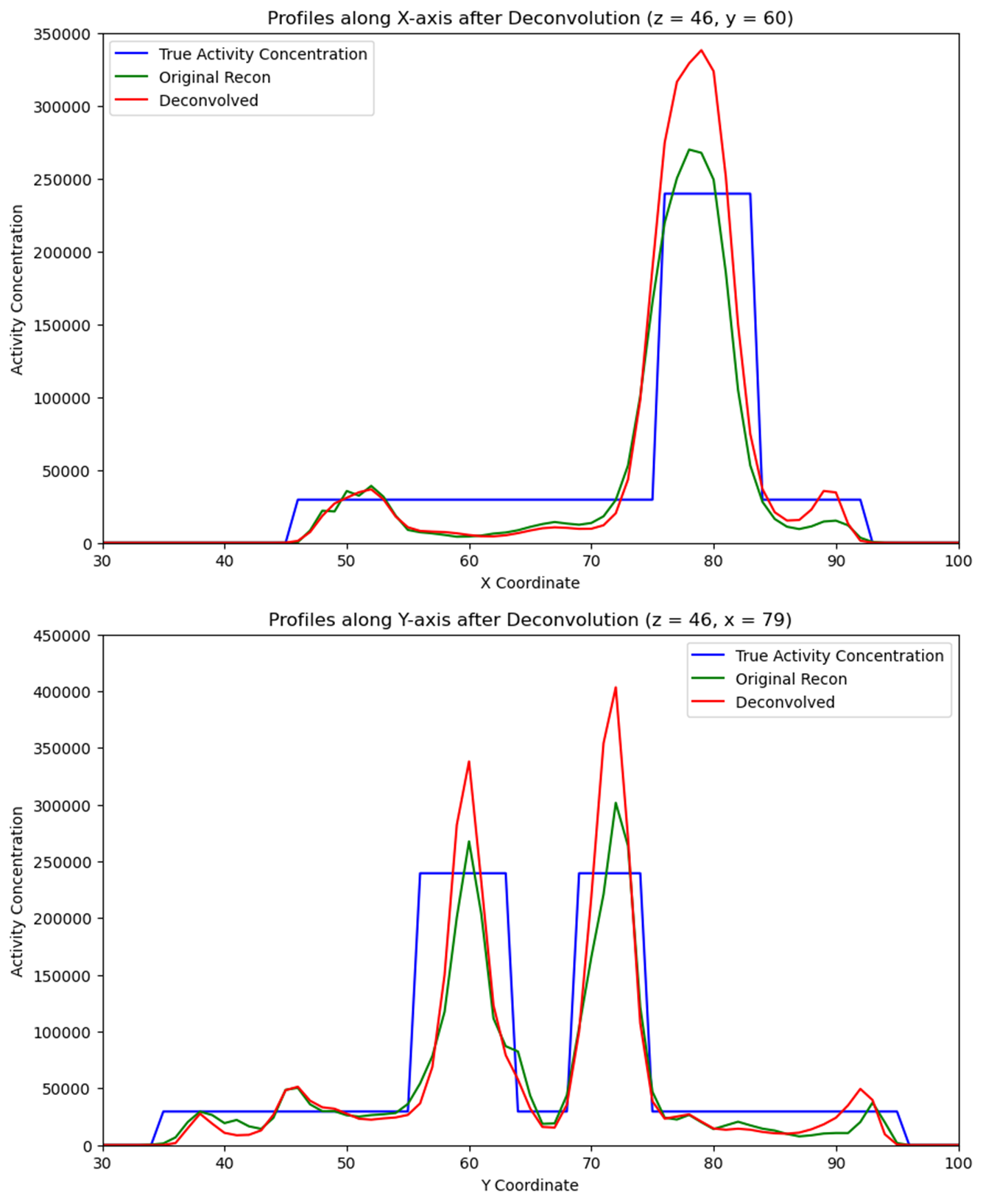


Figure 2: In this phantom based image the SBR was 8:1. LRD parameters were sigma of 6.0 mm and 4 iterations. Top shows the line profile across the x-axis of the image while the bottom shows the line profile across the y-axis of the image.


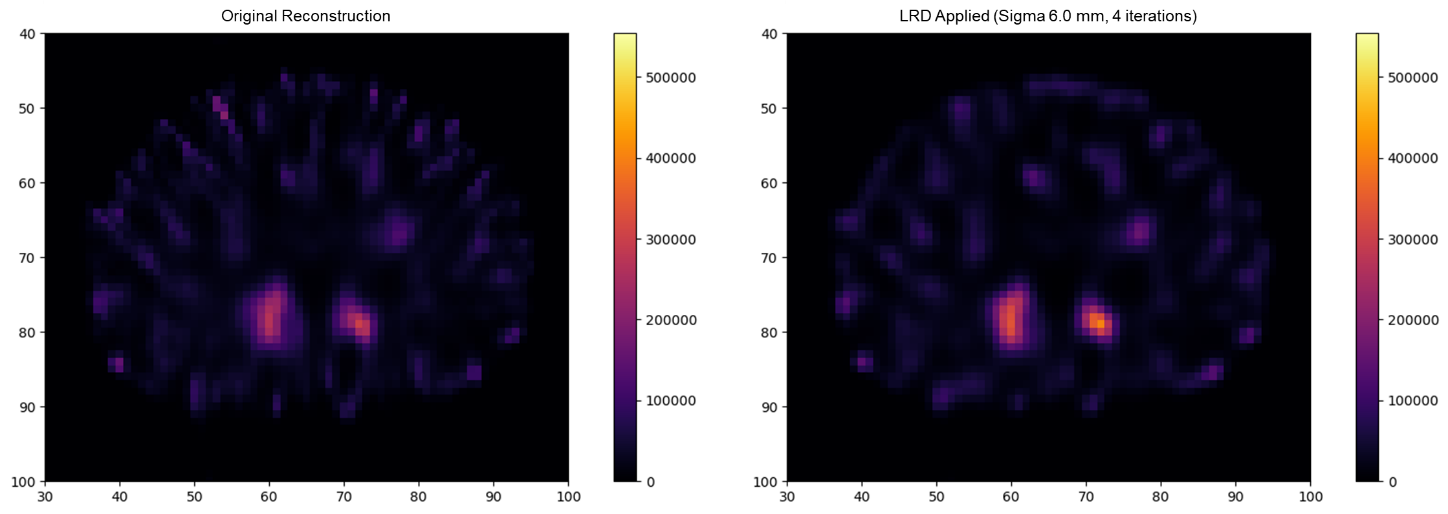


Figure 3: In this phantom based image the SBR was 8:1. LRD parameters were sigma of 6.0 mm and 4 iterations.

The following figures show a more homogenous activity distribution (yellow arrows indicate easily identified areas) (less streaking areas identified using green arrow) in example patients using LRD derived image compared to the Original Reconstruction.

Figure 4: Example sagittal images of a mCRPC patient showing the Original Reconstruction [left] and LRD applied (sigma 6.0 mm, 4 iterations) [right].


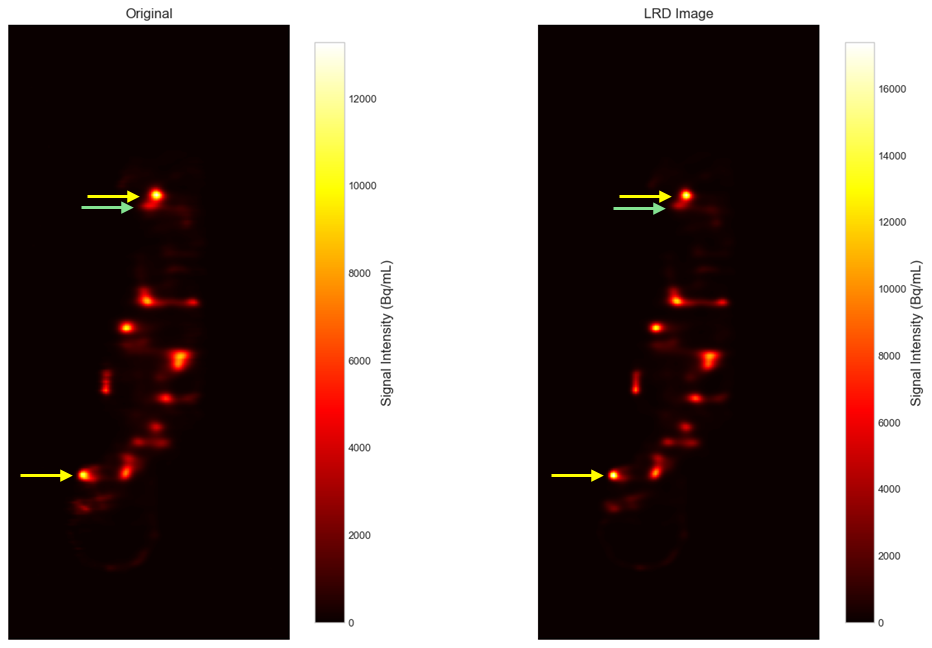


Figure 5: Example coronal images of a mCRPC patient showing the Original Reconstruction [left] and LRD applied (sigma 6.0 mm, 4 iterations) [right].


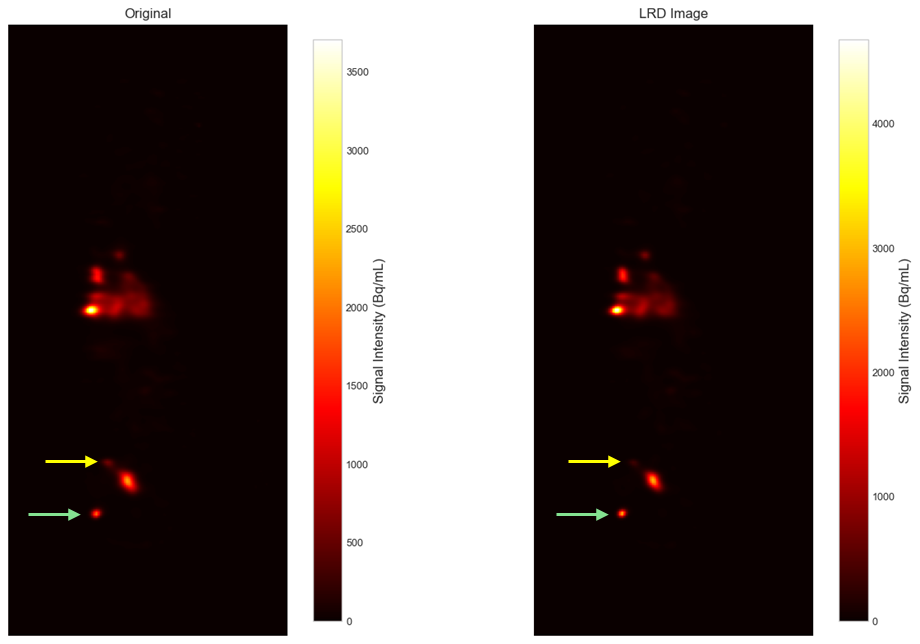

Supplement: Supplementary file 1 — Supplementary Material 1. [file 40658_2026_865_MOESM1_ESM.docx]
